# Diurnal motor activity and “sunbathing” behaviour in crested porcupine (*Hystrix cristata* L., 1758)

**DOI:** 10.1038/s41598-019-50784-y

**Published:** 2019-10-03

**Authors:** Francesca Coppola, Giuseppe Vecchio, Antonio Felicioli

**Affiliations:** 1 Department of Veterinary Science, University of Pisa, Pisa, 56124 Italy; 2 Studio Agrofauna, Livorno, 57121 LI Italy

**Keywords:** Ecology, Evolution, Zoology

## Abstract

The crested porcupine is a mainly nocturnal mammal that shows both moonlight avoidance and some diurnal motor activity, the latter as an extension of its night-time foraging behaviour. Furthermore, a peculiar daytime behaviour, described as “sunbathing”, was reported as episodic in *H. africaeaustralis*. Between 2016 and 2019 a camera-trapping monitoring was performed within 10 porcupine settlements in order to detect the diurnal motor activity and to verify and describe the sunbathing behaviour in crested porcupine. Out of 1,003 trap days, a total of 148 events of diurnal motor activity were recorded. The diurnal motor activity occurred throughout the year mainly between December and June from 15:00 to 16:00, with no statistical difference between cubs, youngsters and adults. The sunbathing behaviour was detected for a total of 36 episodes recorded. Sunbathing was performed mainly by cubs. The sunbathing behaviour occurred only between April and June during the hottest hours of the day (11:00 to 12:00). Diurnal motor activity and sunbathing behaviour of porcupine are discussed in relation to food availability and porcupine physiology.

## Introduction

The temporal activity patterns of wild animals are changing continuously due to climatic conditions, predation, human-related disturbance, food resource availability or a combinations of these^1,2^. Diurnal mammals adapt their activity to darkness or bright moonlight nights in order to reduce the risk of predation, increase the success of predation, reduce energetic cost in terms of thermoregulation, and for foraging strategy^1,3–8^. At the same time some nocturnal animals switch their rhythms of activity, such as feeding and thermoregulation strategy, to daylight^9,10^. Sunbathing (direct exposure to maximum sun) is a common thermoregulation behavioural mechanism both in ecto and endotherms animals that live in desert and arctic areas^11^.

Sunbathing also occurs in a wide range of bird species^12–14^ and it has been frequently observed in primates^15–17^, felines^18^ and some small mammals^19^. Among rodents the sunbathing behaviour is normally exhibited in some small diurnal species such as round-tailed ground squirrel (*Spermophilus tereticaudus*)^20^ and gundi (*Ctenodactylus gundi*)^21^. No data are available for nocturnal rodents. Kingdon^22^ reported for the first time the observation of two adults and two young porcupines “enjoying a siesta” under a shady bush close to a large hole in Africa. Kingdon^22^ did not specify whether his observation concerned *Hystrix cristata* or *Hystrix africaeaustralis*. He has been the first to hypothesise that porcupines do perform a sunbathing behaviour. Yellen^23^ also suggested this behaviour in *H. africaeaustralis*. Subsequently no other observation and data on sunbathing behaviour in other porcupine species of sub-genus *Hystrix* (*H. cristata* and *H. indica*) have been recorded, nor whether such behaviour is specie-specific related. Porcupines are semi-fossorial (they do not live exclusively undergrounds) mainly nocturnal rodents and spend most of the daylight hours in burrows or natural shelters and caves^22,24–26^. The nocturnal activity of porcupines occurs throughout all year and varies according to species. *H. cristata* is mainly active in the first part of the night (from 8:00 p.m. to 11 p.m.) but it also shows high activity in the crepuscular interval^27,28^. Corsini and colleagues^28^ have also observed the presence of an irregular diurnal motor activity of *H. cristata*, throughout the year with a peak in spring. Daylight movements of crested porcupines were usually recorded in the vicinity of a burrow^28^. Crested porcupine shows moonlight avoidance behaviour^25,29–31^ thereby the nocturnal motor activity is significantly lower in bright nights of full moon compared to new moon phases, without any seasonal difference observed^29,30^. *H. indica* is more active in the central part of the night (from 11 p.m. to 2 a.m.) than in crepuscular and daylight periods and no diurnal activity has been recorded^32^. In *H.indica* the moonlight avoidance is maximum in winter and absent in late summer^33^. Few data are available concerning motor activity in cape porcupine (*H. africaeaustralis*). This species seems to be more active in the central part of the night (after the 22:00 p.m.) with a decreasing of the activity towards sunset and very little crepuscular activity^34^. The moonlight avoidance seems not to be present in *H. africaeaustralis*^34^.

In this study we predicted the presence of diurnal motor activity not due to anticipation of emerging and/or delay in returning to the burrow. Moreover part of porcupine diurnal activity, not linked to feeding strategy, such as sunbathing behaviour, is also predicted. This investigation aims to I) detect and measure the diurnal motor activity of three age class crested porcupines in the surrounding of their sett, II) assess the presence as well as describe the sunbathing behaviour in order to contribute to the knowledge of this elusive rodent.

## Results

Video-trapping effort resulted in 1,003 trap days (TD) for a total of 48,441 total videos (TV) collected. The camera-trapping efficacy resulted 48.8% for a total of 23,657 useful videos (UV) where animals were caught on camera. Crested porcupine was detected in 78.8% of UV (n = 18,646 videos). Each monitored settlement was inhabited and/or frequented by a recognisable porcupine family group.

### Diurnal motor activity

**Figure 1:**
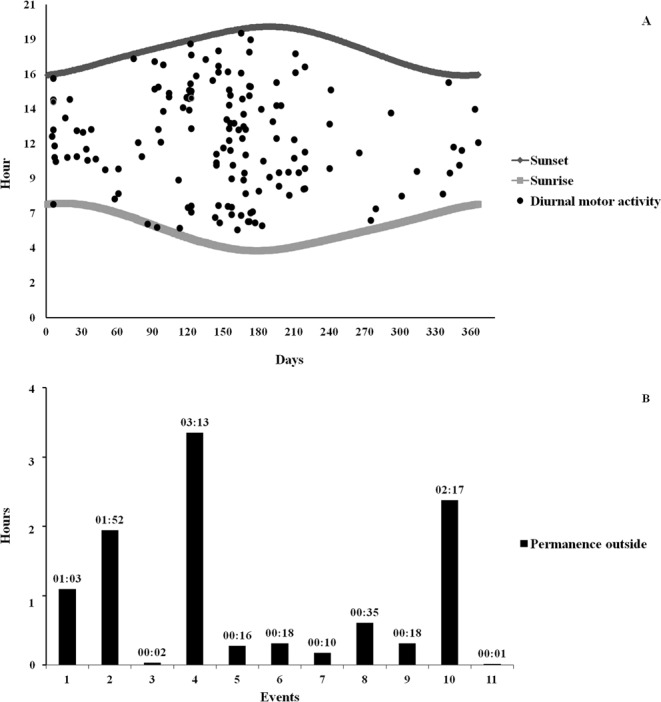
Graphical representation of the 148 events of diurnal motor activity recorded in the monitored settlement in relation to the hour of sunset and sunrise during the year (21 December–20 December).

Diurnal motor activity was recorded in all the monitored settlements. A total of 148 events of diurnal motor activity were recorded in 214 videos (1.1% of videos where porcupines were present) (Fig. 1A). Times of permanence outside the burrow were obtained from 11 diurnal motor activity events (Fig. 1B). The average time of permanence outside the burrow resulted in 55 minutes SD 1 hour and 4 minutes. The minimum permanence time was up to 1 minute and the maximum time up to 3 hours and 13 minutes.

**Figure 2:**
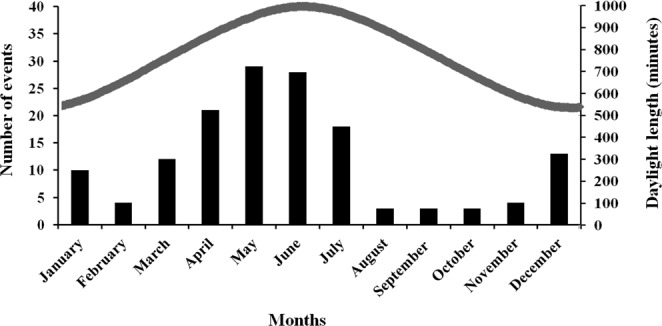
Total number of monthly diurnal motor activity events recorded in the monitored settlement in relation to daylight hours during the year.

**Figure 3:**
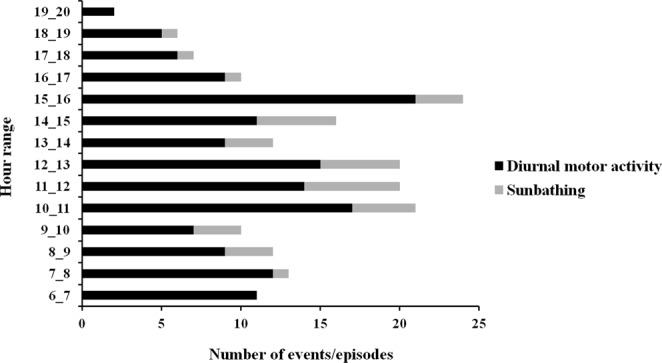
Number of hourly diurnal motor activity events and sunbathing episodes recorded in the monitored settlement during the camera-traps monitoring.

**Figure 4:**
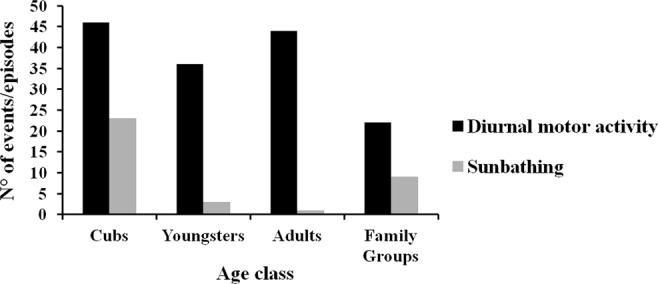
Total number of events of diurnal motor activity and sunbathing recorded in the monitored settlement in cubs, youngsters, adults and family groups.

**Figure 5:**
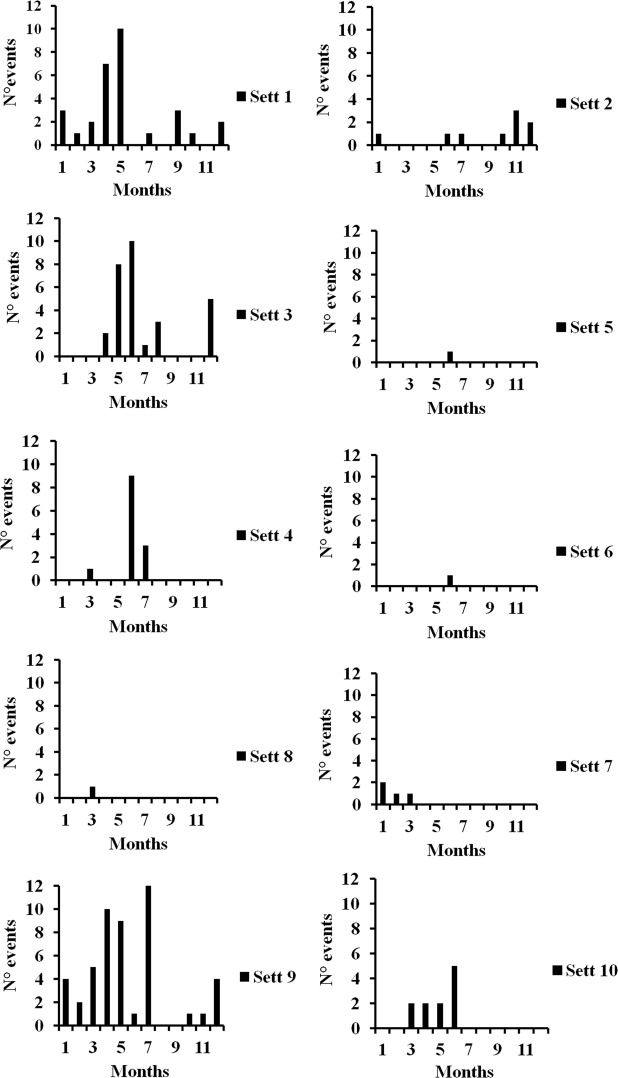
Number of monthly diurnal motor activity events recorded in the settlements permanently (Sett 1, 3, 4, 8, 9) and occasionally (Sett 2, 5, 6, 7 10) inhabited by porcupines.

A diurnal motor activity irregular pattern was observed within each porcupine family throughout the year (Fig. 2). The 92.2% (n = 135 events) of porcupine diurnal movements were recorded between December and July with a peak between April and June (n = 78 events). The 8.7% (n = 13 events) of diurnal motor activity was detected between August and November. The diurnal motor activity was recorded at all hours in daytime with a peak between 15:00 to 16:00 (Fig. 3). The 28.3% of daylight events were performed by cubs (n = 46 events), the 24.3% by youngsters (n = 36 events), the 29.7% by adults (n = 44 events) and the 14.8% by family groups (n = 22 events) (Fig. 4). The diurnal motor activity resulted significantly lower in family groups compared to lonely youngsters (P < 0.05), cubs and adults (P < 0.001). No significant differences were recorded among the cubs, youngsters and adults daylight movements. The occurrence of diurnal motor activity resulted significantly higher in the setts permanently inhabited (P < 0.001) than in those only occasionally inhabited by porcupines (Fig. 5).

### Sunbathing behaviour

**Figure 6:**
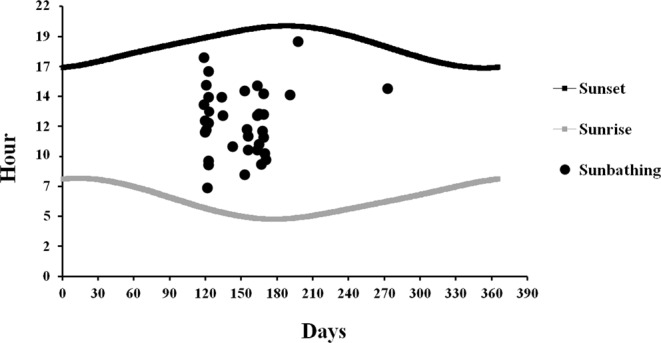
Graphical representation of the 36 episodes of sunbathing recorded in the monitored settlement in relation to the hour of sunset and sunrise during the year (21 December–20 December).

**Figure 7:**
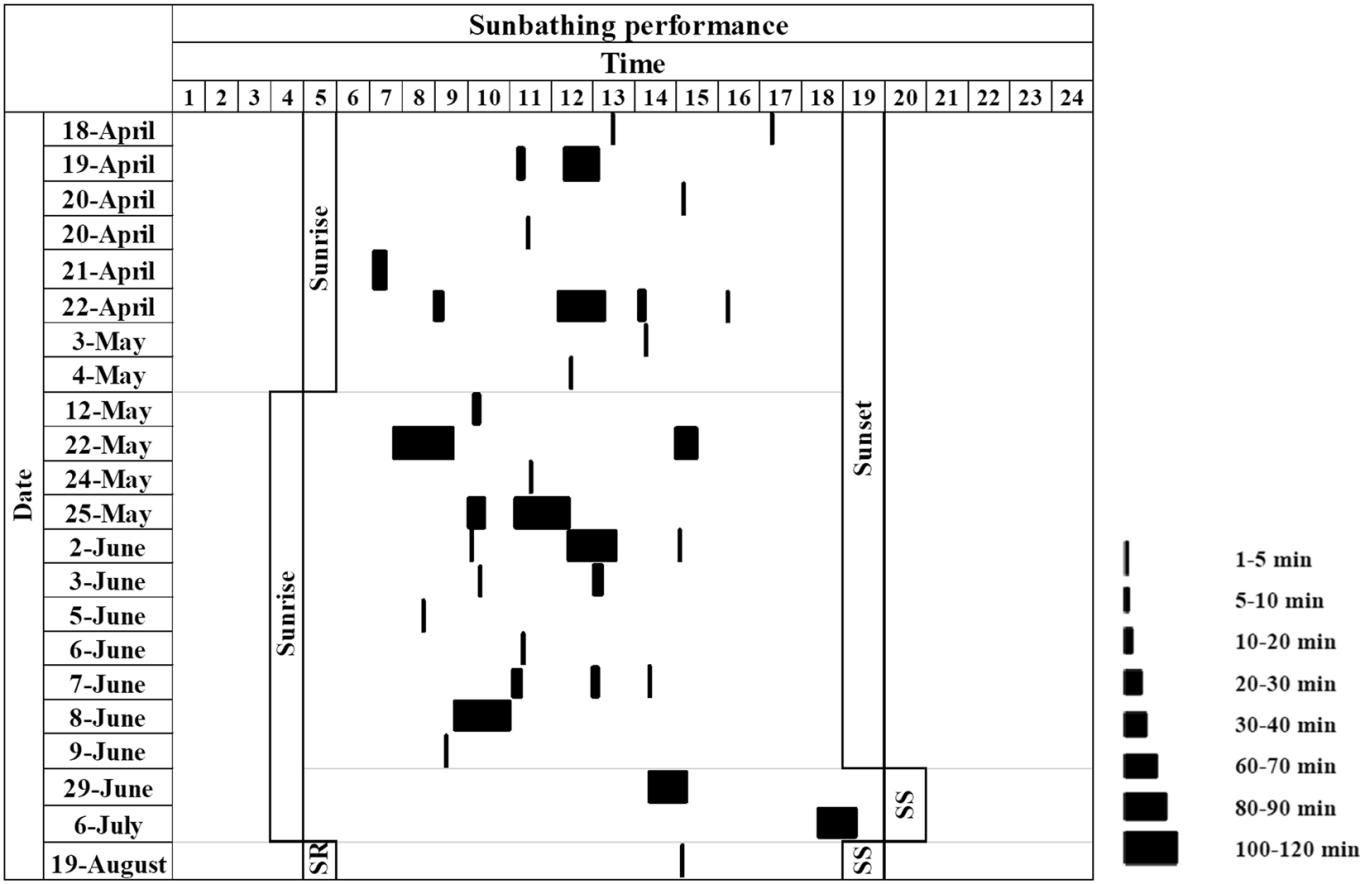
Graphical representation of the length of sunbathing episodes recorded. For each sunbathing episode the date, the hour, the duration (min), the time of sunset (SS) and sunrise (SR) was indicated.

The sunbathing behaviour was detected in 0.8% (n = 160 videos) of videos where porcupines were present for a total of 36 episodes (Fig. 6). The sunbathing was observed only in porcupines belonging to 5 family groups in sett 1, 3, 6, 9 and 10 always in the vicinity of the ground hole entrance of the burrow. In the 64% of recorded episodes the sunbathing behaviour was performed by cubs (n = 23 episodes), in the 8.3% by youngsters (n = 3 episodes), in the 2.7% (n = 1 episode) by adults and in the 25% (n = 9 episodes) by cubs with adults or youngsters of the same family (Fig. 4). The occurrence of sunbathing behaviour was significantly higher in cubs (64%) (P < 0.001) compared to youngsters (8.3%), adults (2.7%) and cubs with adults or youngsters (25%). The sunbathing performance in cubs with adults or youngsters was higher than in youngsters only (P < 0.05) and adults only (P < 0.01). No statistical difference resulted between adults and youngsters sunbathing performances. The 72.2% of sunbathing episodes was recorded between April and June mainly from 11:00 to 12:00 (Fig. 3). The average duration of the sunbath resulted to be 20 minutes SD 28 minutes with the duration range between 1 to 123 minutes (Fig. 7).

## Discussion

Crested porcupine spends most of nocturnal hours searching for food^22,35,36^. Corsini *et al*.^28^ also described the presence of some diurnal activity in the crested porcupine and hypothesised that this behaviour maybe due to an extension of their night-time foraging behaviour or thermoregulation strategy. The diurnal motor activity in crested porcupines was observed in all the monitored settlements and shows an irregular pattern throughout the year. The diurnal motor activity events were recorded mainly between December and June with a peak from April to June in accordance to Corsini and colleagues^28^. The diurnal outdoor permanence ranged from 1 minute to 3 hours and 13 minutes. The increasing of occurrence of the daytime activity between December and June could be due to the combined effect of I) reduction of darkness hours necessary for feeding II) reduction of favourite food due to season changes. Most of porcupines favourite food (roots, bulbs, tubers and rhizomes of many wild and cultivated herbaceous plants) is mainly available in late summer, autumn and early winter^24,25,35–38^.

According to Corsini and colleagues^28^ crested porcupines seem to show an irregular diurnal motor activity with a peak in spring when the night becomes progressively shorter and a lack of diurnal activity during summer nights. These Authors do not explicit whether this behaviour is the result of anticipation of emerging and/or delay in returning to the burrow. The same Authors speculate that this behaviour could be either a feeding and/or thermoregulation strategy. In this investigation the porcupine motor activity was performed throughout all daytime hours with a preference for central hours therefore it cannot be connected to anticipation of emerging or delay of returning to the burrow. In accordance to Corsini and colleagues^28^, these results support that diurnal motor activity could be linked to feeding strategy. The presence of diurnal motor activity also in summer days has been observed thereby excluding thermoregulation needs, as hypothesised by Corsini and colleagues^28^. The Italian population of crested porcupines seems to have African origins^39^ where this species lives in desert areas of northern Africa and Sub-Saharan Africa overlapping its distribution range with *H. africaeaustralis*^22^. Study performed in captivity shows that cape porcupines (*H. africaeaustralis*) acclimated to 25 °C can regulate their body temperature between 13° and 30 °C^40^. No data are available for both crested porcupine (*H. cristata*) and *H.indica*. However it can be assumed that also *H. cristata* may have similar ranges of thermoregulation. Moreover no information is available concerning the thermoregulation ability of this species at low temperatures. In Italy porcupines live in temperate areas with hot summers and mild winters^41,42^. Therefore it is more likely that these environmental conditions can be widely tolerated by porcupines without significant thermoregulation need.

Family group daylight movements resulted significantly different from lonely specimens of all ages. On the other hand, no difference was recorded in diurnal motor activity between lonely specimens (cubs, youngsters or adults). This result suggests that daylight behaviour could not be related to sub-adults dispersion although no information is available on the dispersal of sub-adults. The only information available is that the youngsters remain with the parents until they reach the adult weight size (10 kg, from 1 to 2 years old)^43,44^.

Diurnal motor activity resulted significantly higher in the setts permanently inhabited by porcupines while sporadic diurnal transits were recorded in settlements occasionally inhabited. This result may exclude a connection between diurnal motor activity and exploration of the environment and thereby supporting the linkage of diurnal movements to feeding strategy.

The sunbathing behaviour was observed in five porcupine families. 32 out of 36 episodes show only cubs or cubs with adults or youngsters. These observations confirm that porcupines perform sunbathing, as hypothesized by Kingdon^22^ and suggested by Yallen^23^, and prove for the first time the presence of sunbathing behaviour in crested porcupine. The sunbathing behaviour seems to be somehow related to the presence of cubs. The presence of adults and youngsters of the same family with the cubs during sunbathing may be due to cubs protection. So it is possible to hypothesise that the cubs need to perform sunbathing for thermoregulation and/or synthesis of metabolites such as vitamins (Vitamin D) necessary for growth. As hypothesised for diurnal motor activity, thermoregulation may not be a necessity for adults. However, considering the lack of information on the ability of thermoregulation of the species it cannot be excluded that the cubs may have some difficulties in thermoregulation. The Vitamin D is necessary for the normal skeletal development in the young as well as maintenance of calcium and phosphate homeostasis in the adults^45^. Reduction in growth and bone deformities were reported to be the main consequences of vitamin D deficiency in diets or of lack of natural sunlight in young animals^46–48^. Exposure to sunlight initiates the formation of vitamin D and studies performed on ponies^47^ and primates in zoos^48^ reported that dietary vitamin D is not needed for normal bone development when there is abundant sunlight. No data are available concerning vitamin D requirement in nocturnal mammals and among these, porcupines. In this investigation sunbathing behaviour was recorded between April and June mainly in the hot hours of the day. The sunlight exposure in hottest period of the year and during hottest hours of the day could reinforce the hypothesis of sunbathing as thermoregulation strategy or sunlight absorption for Vitamin D production. For all diurnal events/episodes, the porcupines showed no changes in their usual nocturnal activity pattern the previous and successive nights. In this case the permanence of porcupines near the burrow during daylight hours seems to be probably due to metabolic necessities. In conclusion the crested porcupine seems not to be a strictly nocturnal mammal. Porcupines show peaks of diurnal activity, probably as feeding strategy, mainly in late winter and spring when the hours of darkness necessary for feeding decrease and there is a reduction of food sources preferred by porcupines. Crested porcupine cubs perform sunbathing in spring. The permanence under sunlight seems to be a behaviour probably regulated by metabolic necessities.

At the light of the results obtained in this investigation, feeding strategy is not the exclusive reasons of the occurrence of diurnal activity in porcupine as predicted in this study. Further investigations concerning the pattern of diurnal activity and physiological aspects of the species are desirable in order to explain the diurnal behaviour of this nocturnal rodent.

## Methods

### Study area and camera-trapping

The investigation was performed in a hilly area of 1,437 ha in Crespina-Lorenzana (43.35412°N; 10. 325052°E) in the province of Pisa (Tuscany, Central Italy) at 86 m a.s.l. The study area is characterized by small black locust (*Robinia pseudoacacia*) and oak (*Quercus cerris*) woods mixed with marginal areas, cultivated areas, olive groves and vineyards. The area is also characterized by a wide variety of wildlife mammals such as the crested porcupine (*Hystrix cristata*), European hedgehog (*Erinaceus europaeus*), wild boar (*Sus scrofa*), roe deer (*Capreolus capreolus*), pine marten (*Martes martes*), stone marten (*Martes foina*), European badger (*Meles meles*), wild rabbit (*Oryctolagus cuniculus*), European hare (*Lepus europeaus*), red fox (*Vulpes vulpes*), and wolf (*Canis lupus*).

The climate is temperate with hot summers (average 23.1 °C in August) and rainy and mild winters (average 6.8 °C in January). The average annual rainfall is 842 mm with peaks between October and December^49,50^. In the study area the crested porcupines are present with a stable population and 46 settlements are known.

Camera-trapping monitoring was performed between November 2016 and July 2019 in 10 out of 46 settlements in the study area. The ten settlements were selected in order to obtain: I) five setts permanently inhabited by porcupines, Sett 1 (180°S), Sett 3 (145°SE), Sett 4 (270°W), Sett 8 (340°NW), Sett 9 (290°NW) and II) five setts only occasionally inhabited by porcupines, Setts 2 (100°E), 5 (90°E), 6 (230°SW), 7 (265°SW), 10 (160°SE).

All the 10 settlements were located in woody areas no more than 60 meters far from feeding areas, ranging from 35 to 60 m a.s.l. Each monitored settlement was known to be inhabited and/or frequented by a recognizable porcupine family. The recognition of each porcupines family was possible due to the presence of adults and sub-adults specimens individually marked in a previous study or recognisable by the presence of phenotypic peculiarities (e.g. blindness, presence of injuries). Twenty camera-traps (Num’axes PIE1009) with passive infrared sensor (PIR) were used. The camera-traps were set to record 20 s long video clips without time-lapse. Each settlement was continuously monitored with two camera traps for the whole monitoring period. Video trapping effort was indicated as number of traps days (TD), while video trapping efficacy was expressed as percentage of total number of useful videos in which animals were present. The camera-trap video recordings were checked and filed on a weekly basis.

### Diurnal motor activity and sunbathing analysis

The diurnal motor activity and sunbathing were investigated within three porcupine age classes: cubs (<5 month old), youngsters (5 to 12 month old) and adults (>12 month old). The porcupine youngsters included juveniles (5 to 8 month old) and sub-adults (8 to 12 month old). In order to distinguish cubs from juveniles we considered the weight and body length parameters reported in Table 1. The sub-adults and adults, due to very similar body size, were recognized by individual markings or phenotypic peculiarities (e.g. blindness, presence of injuries).

**Table 1:** Age range (month), weight (Kg) and body length (cm) parameters for each porcupine age class used to assess the age class of porcupine recorded in camera-traps.

Age class	Age range	Weight	Body length
Cubs	<5	<5	10–30
Juveniles	5–8	5–8	30–60
Sub-adults	8–12	9–11	60–90
Adults	>12	12–15	60–90

Diurnal motor activity was assessed when returning to or emerging from the burrow and when transits of porcupines in the settlements area during daylight hours were observed. All those videos recorded during daylight time from 60′ after sunrise to 60′ before sunset were considered for the analysis of diurnal motor activity. In addition those videos of emerging from burrow starting from 30′ before sunrise as well as all those of returning to the burrow within 30′ before sunset, were also included. Consecutive videos clearly attributable to the same specimen and/or family group were considered as a single event. All events of diurnal motor activity in relation to sunrise and sunset times throughout the year were recorded in a graph. For each diurnal event the date, time and age class of detected porcupines were reported and, where possible, the outside permanence was also calculated. The frequency of diurnal motor activities in the setts permanently and occasionally inhabited were analysed using chi square test (χ^2^).

Sunbathing behaviour was assessed by recording porcupines pausing in front of the burrow for at least 1 minute. Even in this case consecutive videos clearly attributable to the same specimen and/or family group were considered as a single episode. The length of each sunbathing episode was established considering the hour of the first and the last observation of the same specimens and/or family group. For each sunbathing episode the settlement, date, time, duration, number and age class of detected porcupines were reported. The frequency (number of events/episode for each porcupines age/total numbers of events/episode) of diurnal motor activity and sunbathing in different porcupine age were analyzed using chi-square test (χ^2^).

## Data Availability

The datasets generated and analysed during the current study are available from the corresponding author on reasonable request.
